# Network Dynamics Caused by Genomic Alteration Determine the Therapeutic Response to FGFR Inhibitors for Lung Cancer

**DOI:** 10.3390/biom12091197

**Published:** 2022-08-29

**Authors:** Jonghoon Lee, Sea Rom Choi, Kwang-Hyun Cho

**Affiliations:** Department of Bio and Brain Engineering, Korea Advanced Institute of Science and Technology (KAIST), 291 Daehak-ro, Yuseong-gu, Daejeon 34141, Korea

**Keywords:** FGFR, lung cancer, systems biology, network science, cancer biology, RTK, precision medicine, signaling pathway

## Abstract

Recently, FGFR inhibitors have been highlighted as promising targeted drugs due to the high prevalence of FGFR1 amplification in cancer patients. Although various potential biomarkers for FGFR inhibitors have been suggested, their functional effects have been shown to be limited due to the complexity of the cancer signaling network and the heterogenous genomic conditions of patients. To overcome such limitations, we have reconstructed a lung cancer network model by integrating a cell line genomic database and analyzing the model in order to understand the underlying mechanism of heterogeneous drug responses. Here, we identify novel genomic context-specific candidates that can increase the efficacy of FGFR inhibitors. Furthermore, we suggest optimal targets that can induce more effective therapeutic responses than that of FGFR inhibitors in each of the FGFR-resistant lung cancer cells through computational simulations at a system level. Our findings provide new insights into the regulatory mechanism of differential responses to FGFR inhibitors for optimal therapeutic strategies in lung cancer.

## 1. Introduction

With the rapid accumulation of the clinical data of patients, precision medicine has emerged as one of the most promising approaches to tackling various diseases, including cancer, by developing therapeutic strategies and improving treatment effectiveness [1]. Despite its wide application in the development of cancer treatment, precision medicine is bound to face some challenges in attaining its ambiguous goal to target the right treatment at the right patient. Due to heterogeneity within cancer patients, tailoring therapies to groups of patients according to their genetic profiles and environments is not as successful for some patients since there is no “one-size-fits-all” therapeutic approach that suits the “average patient” [2]. Thus, drug efficacy and therapeutic responses of patients vary even though they share similarities in genetic profiles that led them to be categorized for the same therapeutic treatment.

Researchers have speculated that this is due to complex interactions between genes and molecules within various signaling pathways that are responsible for drug sensitivity. One example would be the fibroblast growth factor receptor (FGFR) signaling pathway which determines major cellular phenotypes, including proliferation, apoptosis, and growth arrest in various cancers such as lung cancer [3,4,5]. Recent studies showed that more than 20% of these patients have copy number alteration (CNA) of FGFR, and inhibiting it was considered a promising strategy to treat them [6]. Moreover, gene amplification and overexpression of FGFR are considered promising biomarkers for anti-FGFR targeted therapy, and clinical trials of several FGFR inhibitors based on these biomarkers are underway [7,8]. However, early-phase clinical trial results reported that patients were only partially responsive to the drugs [9,10]. Studies have suggested that this is due to complex regulatory interactions of various downstream molecules within the FGFR cascade such as RAS/RAF/MEK/ERK, PI3K/AKT, PLCgamma/PKC, and JAK/STAT [11,12,13]. In addition, heterogeneous genetic alterations in patients can rewire the intracellular molecular interactions that can cause major changes in the dynamics of various signaling pathways, which can result in differential responses to FGFR inhibitors [14,15]. Therefore, there are limitations in using conventional treatment strategies based on biomarkers to treat a heterogeneous population of lung cancer patients without any mechanistic understanding of drug sensitivity.

Systems biological analysis of network dynamics using quantitative mathematical models can allow us to predict heterogeneous drug responses of cells according to their various genomic conditions. Molecular interactions within a cell are often represented as network models with their molecular components as nodes (proteins, genes, and small molecules) and interactions as edges [16]. A well-represented network model can be used to recapitulate complex dynamics of biological phenomena and to predict cellular behaviors under certain conditions [17,18]. To analyze such network models with complex dynamics, a Boolean model with binary node states can be used to represent each of the network components in a discrete way; ON (activation) as one and OFF (inactivation) as 0. Unlike other continuous mathematical modeling with ordinary differential equations, the Boolean model does not require any kinetic parameters which makes it possible to avoid various problems in parameter estimation [19,20]. Moreover, signaling cascades and state transitions between node states are traceable after applying certain perturbations which allow one to analyze differential network dynamics associated with drug efficacy in patients with heterogeneous genomic conditions. Thus, we can overcome the limitations of precision medicine by investigating individualized complex mechanisms of the drug effect through a systems biological approach.

In this study, we aim to understand the underlying mechanisms of heterogeneous responses to FGFR inhibitors and identify potential targets that can induce optimal therapeutic responses. For this, we have reconstructed Boolean network models for FGFR/FGF aberrant lung cancer cell lines with different genomic conditions and investigated the fundamental mechanisms of differential responses to FGFR inhibitors from each model. Moreover, we performed extensive perturbation analysis using these network models to identify optimal targets that can induce therapeutic responses to FGFR inhibitors or other alternative drugs according to their genomic conditions and elucidate underlying mechanisms from each of our models. Our study provides insights into a mechanism-based therapeutic strategy by using a dynamical network model to increase the effectiveness of various targeted therapies for cancer.

## 2. Materials and Methods

### 2.1. Input-Output Relationships of the Lung Cancer Network Model

In a Boolean network model, the state of each node represents the activity of a signaling protein in a simple way, that is, 1 (ON) for an active state or 0 (OFF) for an inactive state without detailed kinetic information [20,21]. In addition, the regulatory effects of input nodes on their corresponding output nodes are modeled based on logical rules that use Boolean operators, “OR”, “NOT”, and “AND”. [22]. To analyze input–output relationships in the model, we performed qualitative simulations with different input frequencies from 0 to 1. The input of interest is fixed as ON during simulation at a specified frequency. We found that our lung cancer network model reached a stable attractor state within 2000-time steps from the sufficiently sampled initial states. Thus, we updated our Boolean functions for 3000 times with each input frequency, and then nodes’ steady-state activity was calculated as the average activity over the last 300 steps.

### 2.2. Selection of Functional Genomic Alterations

Genomic information of lung cancer cell lines from the Cancer Cell Lines Encyclopedia (CCLE) project, including somatic mutation, CNA, and messenger RNA (mRNA) expression, were obtained from cBioportal (https://www.cbioportal.org/study/summary?id=ccle_broad_2019, accessed on 26 March 2022). We selected seven cell lines (NCIH1581, NCIH1781, NCIH520, NCIH1703, LK2, HCC15, and NCIH810) that have highly amplified expressions of FGFR1-3 and FGF with the available drug response data for AZD4547 or PD170374 from the GDSC2 database. To selectively choose the genes with functional genomic alteration, we analyzed the data as follows (refer to Supplementary Figure S1 for details): If a gene is included in our network model and has a somatic mutation with gain-of-function (GOF) from OncoKB databases, then we set the activity of this gene as ON (1). If a gene has a somatic mutation with loss-of-function (LOF), then we set the activity of this gene as OFF (0). In addition, variant_classification from the maf file is annotated as ‘Nonsense_Mutation’, ‘Nonstop_Mutation’, ‘Frame_Shift_Ins’, or ‘Frame_Shift_Del’, we also set the activity of the gene as OFF. For mRNA expression, z-score normalized mRNA expression data were downloaded from the Cancer Cell Lines Encyclopedia (CCLE) cohort [23]. We set a gene as overexpressed if its z-score is >−2 or underexpressed if its z-score is <−2. For CNA, we set a gene as amplified (AMP), if its GISTIC2 score is 2 and its mRNA expression is >1. Moreover, we set a gene as deletion (DEL), if its GISTIC2 score is −2 and its mRNA expression is <−2.

### 2.3. Mapping Genomic Alterations to Network Model

To construct a differentially wired network model for each cell line, the functional genomic profiles of the cell line were mapped onto the edges and nodes of the nominal model. For input node, the mRNA expression level was used to set a continuous value. For the rest of the nodes, functional genomic alteration was used to set the state of each node in the network model. Because several genes could correspond to the same node in our network model, we determined the priority among variants for each gene. In the case of mutation, if any of the genes that were mapped onto the same node has a GOF mutation, we set it as GOF. If all of the genes that were mapped onto the same node have LOF mutations, we set it as LOF. We set the rest as neutral. For CNA, if any of the genes mapped onto the same node has AMP or DEL, we set the node as AMP or DEL, respectively. The rest were set as diploid. Finally, for mRNA expression, if any of the genes that were mapped onto the same node were overexpressed, the node was set as overexpressed. If all of the genes were underexpressed, then the node was set as underexpressed. The rest were set as normal expression. Then, for nodes other than the input node, we used the functional genomic profiles of the cell lines and applied them onto our Boolean model as follows:

For the case of mutation, nodes with GOF or LOF were fixed as permanent ON or OFF, respectively. For the case of CNA and mRNA expression, the weight of all negative links from the nodes with AMP or overexpression was set to 0 in order for these nodes to be more effectively activated by their upstream nodes. Conversely, for the case of DEL and underexpression, all positive incoming weights were set to 0 to prevent the activation of the corresponding nodes. For input nodes, we further calculated the input strength for each input node within a cell. First, when multiple genes were mapped to one node, we first chose the maximum z-score of those genes. Then, we transformed it to a continuous value between 0 and 1 by using a sigmoid function(1/(1 + exp(−(x − k)), k = 3 in this study. We defined the value as input strength of the corresponding node. If an input node had a mutation, we set the input strength of it as 1 for GOF or 0 for LOF. The schematic representation of creating the functional genomic profile is described in Supplementary Figures S1 and S2.

### 2.4. Defining Cell Line-Specific Initial State Probability

We defined the cell line-specific initial state probability for each node as 1 according to its corresponding functional alteration profiles. For the nodes other than input, if their genomic profiles have either overexpression or AMP, the probability was set to 1; if they have DEL or underexpression, they were set to 0; and the remaining nodes were set to 0.5. For input nodes, the probabilities were set to their corresponding input strength. For example, if the input strength of an input node is 0.9, then it has a 90% chance of having ON states and a 10% chance of having OFF states as its initial condition for that input node. Based on the defined probability, we randomly generated 20,000 initial states for attractor landscape analysis.

### 2.5. Attractor Landscape and Perturbation Analysis Using the Boolean Network Model of Lung Cancer

Our lung cancer network model has 2^56^ possible states that consist of the entire states of the network model with 56 nodes without three output nodes. We randomly sampled 20,000 initial states according to cell line-specific probability for each node (refer to *Defining cell line-specific initial state probability* for details), which was a sufficient number to cover the major attractors. Network dynamics of each cell were analyzed using attractor landscape, which consists of trajectories from the sampled initial states of the cell to its attractor states through synchronous updating schemes. Then, we calculated average node activity (*A*) for each node as follows:

$$\begin{array}{cc} Average\;node & activity\;\left(A\right)\\ & =\overset{attractors}{\underset{i}{\sum }}(ON\;frequency\;of\;a\;node\;in\;the\;attractor\;i\\ & \times basin\;ratio\;of\;the\;attractor\;i) \end{array}$$

To quantify results from the network perturbation analysis, we derived a phenotype score (*P*) for each cell line in a specific simulation setting, including control and each perturbation, based on the average activities of phenotype nodes as follows:

*Phenotype score* (*P*) = *A_P_* − *A_A_* − *A_G_*

where *A_P_* stands for the average node activity of Proliferation, *A_A_* for the average node activity of Apoptosis, and *A_G_* for the average node activity of Growth_arrest. Based on the phenotype score, we calculated the drug response score (*D*) for each cell line as follows:

*Drug response score* (*D_drug_*) = *P_drug_* − *P_control_*

where *P_control_* represents the phenotype score before drug perturbation simulation and *P_drug_* represents the phenotype score after drug perturbation simulation.

### 2.6. FGFR Inhibitor Response Data

Area under the curve (AUC) values of AZD4547 and PD173074 from the GDSC 2 for all cancer cell lines were downloaded from DepMap 22Q1 (https://depmap.org/portal/download/, accessed on 16 May 2022). The AUC matrix was transformed to a gene-wise robust z-score and we took a subset of the seven cell lines that have genomic alterations in FGFR1-3 or their cognate ligands.

## 3. Results

### 3.1. Network Dynamics-Based Drug Response Prediction

**Figure 1:**
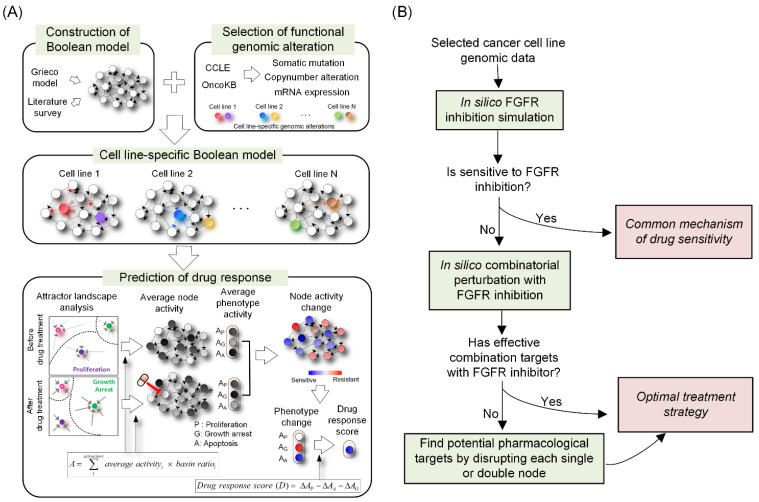
Network dynamics-based cell line-specific drug response prediction model using attractor landscape analysis. (**A**) We gathered data from a Boolean model (Grieco et al., 2013 [24]) and signaling pathways identified from the literature to build a lung cancer-specific Boolean network model. The functional genomic alterations of cancer cells are mapped onto our nominal network model to create differentially wired network models that have distinct network topologies. To predict cell specific drug responses, we conducted systematic perturbation and then analyzed their attractor landscape to calculate average node activity. The final drug response score of each cell line to particular drug is obtained based on the difference between phenotype scores before and after perturbation. (**B**) The procedure of identifying optimal targets from each cell line-specific network model.

Our systems biological approach to predict cell-specific responses consists of four major steps as follows: (1) construction of a Boolean model by extending a generic Boolean cancer network model (Grieco et al., 2013 [24]), which integrates major signaling pathways and their substantial crosstalks; (2) selection of functional genomic alterations from the CCLE cohort; (3) construction of cancer-specific Boolean models based on functional genomic alterations in cancer cell lines; and (4) evaluation of drugs’ efficacy in predicting cell line-specific responses based on perturbations of each cell line-specific network model (Figure 1A). We then followed the procedure shown in Figure 1B to identify potential pharmacological targets and their mechanism in regulating drug sensitivity. The workflow is summarized in Figure 1.

### 3.2. Construction of a Network Model of Lung Cancer

**Figure 2:**
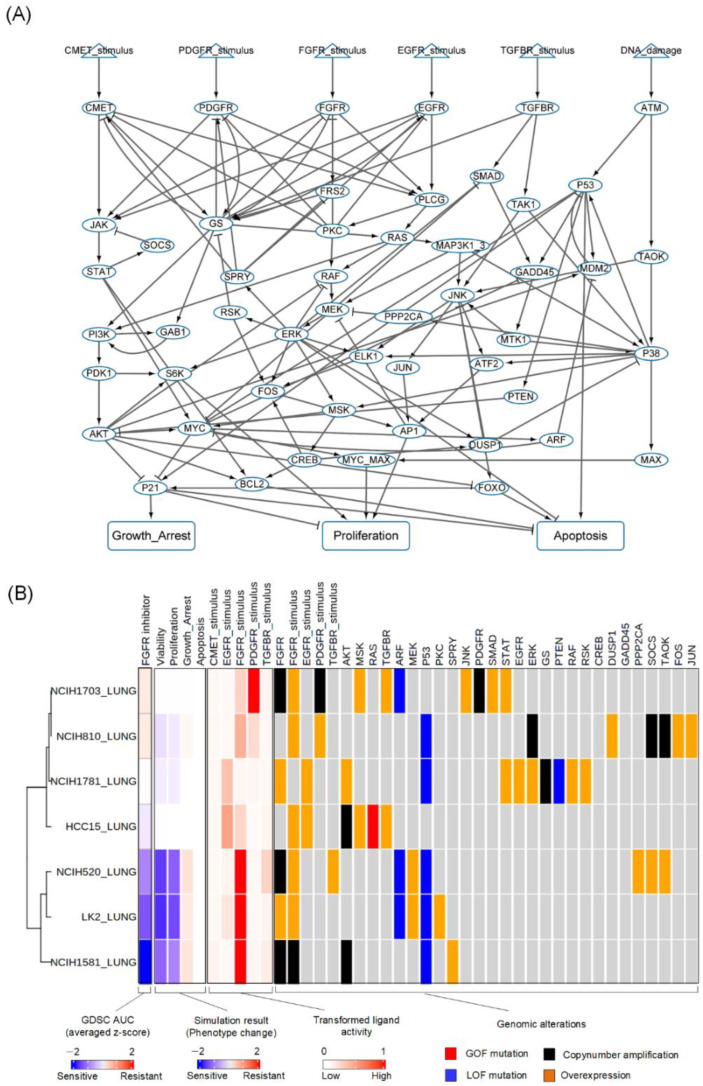
Reconstruction of lung cancer network model with cell line-specific genomic information and their predicted drug responses to FGFR inhibitor. (**A**) Our constructed lung cancer network with 59 nodes and their 138 molecular interactions created using public databases. There are a total of six nodes for input signals, including FGFR_stimulus, EGFR_stimulus, TGFBR_stimulus CMET_stimulus, PDGFR_ stimulus, TGFB_stimulus, and DNA damage. There are three output nodes, including proliferation, apoptosis, and growth arrest. (**B**) Seven cell line-specific lung cancer network models are shown. Responses of the corresponding cell lines from the GDSC to FGFR inhibitors are shown in the first column. Simulation results of the predicted responses to FGFR inhibitor are shown in the next column. Genomic alteration information and activities of the network components from the corresponding cell line-specific network models are shown.

There are complex interactions within various signaling pathways of lung cancer cells underlying the regulatory mechanism that leads to resistance to FGFR inhibitors. To comprehensively understand this mechanism, we have constructed a prior-knowledge network model based on a generic cancer Boolean network model [24]. Thus, our model incorporates major signaling pathways associated with the FGFR signaling cascade and their extensive crosstalk determining cellular phenotypes, including proliferation, growth arrest, and apoptosis. We further extended the model with other RTKs such as hepatocyte growth factor receptor (CMET) and platelet-derived growth factor receptor (PDGFR) as well as their downstream signaling molecules involved in the JAK/STAT pathway [25,26,27]. As a result, our network model consists of 59 nodes and 137 links, including six input nodes and three output nodes (Figure 2A). The input nodes of our model represent six external stimuli: fibroblast growth factor (FGFR_stimulus), epidermal growth factor (EGFR_stimulus), hepatocyte growth factor (CMET_stimulus), platelet-derived growth factor (PDGFR_stimulus), transforming growth factor-beta (TGFB_stimulus), and DNA damage (DNA_damage). Moreover, these stimuli affect the corresponding signaling cascades related to FGFR inhibitory responses. These stimuli regulate the following pathways that are interconnected within our network model: the mitogen-activated protein kinase (MAPK), phosphoinositide 3-kinase (PI3K)/AKT, JAK/STAT, TGFB, TP53, DNA damage-related ATR/ATM, and p38/JNK pathways. Our network model represents molecular interactions between the FGFR and other RTK signaling pathways that govern cellular responses to FGFR inhibitors. To qualitatively validate the constructed network model, we analyzed the input–output relationship. To do this, we conducted in silico simulation by varying input levels from 0% to 100% to examine how well the network model could replicate the biological properties of signaling cascades in lung cancer cells. Through this, we can confirm that a network model is qualitatively validated to reproduce such biological phenomena by evaluating the simulation results according to the levels of incoming signals. We compared our data with previous experimental results. The simulation results after inducing FGF exhibited a positive relationship with its downstream nodes such as ERK, AKT, S6K, and MYC (Supplementary Figure S3). These results indicate that our network model can reflect the biological properties of lung cancer cells.

### 3.3. Reflecting Molecular Features of Lung Cancer Cell Lines to the Network Model

Prior to computational simulation, we reconstructed our network model to cell line-specific networks by integrating genetic alterations of lung cancer cell lines from the CCLE cohort. For this, we selected lung cancer cell lines with amplified or overexpressed FGFR1-3, or any of the FGF family that were overexpressed. Among the selected cell lines, we chose the cell lines with available drug response data of FGFR1-3 inhibitors such as AZD4547 and PD173074. We selected a total of seven cell lines and applied the functional genomic profile of each cell line onto our lung cancer network to create differentially wired network models that represent the genomic landscape observed in distinct cancer cells (Figure 2B and Supplementary Figure S2).

### 3.4. Prediction of Cell Line-Specific Drug Responses to FGFR Inhibitor

To investigate cellular responses to FGFR inhibitors, we performed an attractor landscape analysis using our differentially wired network models of the corresponding cell lines. We compared the simulation results with FGFR inhibitor response datasets of the selected lung cancer cell lines from the GDSC. As a result, our predicted drug responses to FGFR inhibitors of the corresponding cell lines were concordant with the public databases. We then performed a clustering analysis, and the cell lines were categorized into sensitive and resistant groups (Figure 2B).

**Figure 3:**
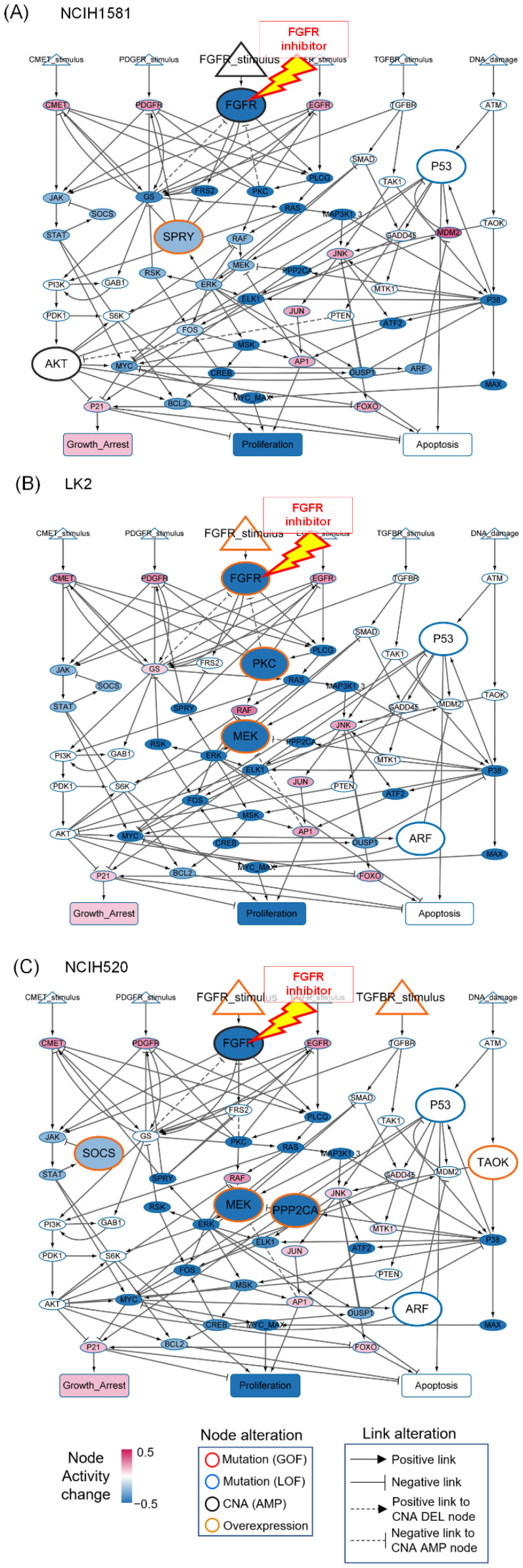
Responses to FGFR inhibitor from the sensitive cell line-specific network models. The average activity changes of FGFR, its downstream molecules, and changes in phenotype nodes before and after the drug treatment are shown for (**A**) NCI-H1581, (**B**) LK2, and (**C**) NCI-H520. A red open circle represents gain-of-function mutation, a blue open circle represents loss-of-function mutation, a black open circle represents copy number amplification, and a yellow open circle represents overexpression. A solid arrow indicates a positive link whereas a blunted-end head arrow indicates a negative link. A dotted arrow indicates a positive link to node with CNA deletion whereas a dotted arrow with blunted end indicates a negative link to node with CNA amplification.

In FGFR inhibitor-sensitive cell lines such as NCI-H1581 (Figure 3A), LK2 (Figure 3B), and NCI-H520 (Figure 3C), inhibition of FGFR led to suppression of the active FGFR and its downstream molecules, including FRS, ERK and MYC, and induced P21, which ultimately decreased proliferation and induced growth arrest. Although these cell lines have different genomic alterations, they all have exclusively high levels of FGFR_stimulus relative to resistant cell lines (Figure 3). Moreover, LK2 and NCI-H520 cell lines have overexpressed MEK which ultimately transmits signaling flow of highly activated FGFR through the ERK pathway. Therefore, we hypothesized that these genomic characteristics shared between the cell lines lead them be more dependent on the FGFR-ERK signaling pathway for their growth, and thus they are sensitive to FGFR inhibitors.

### 3.5. Resistant Responses to FGFR Inhibitor from the Representative Cell Line-Specific Network Models

In FGFR resistant cell lines, FGFR inhibition does not affect the activity of downstream molecules in the FGFR signaling cascade. Interestingly, these cell lines also have distinct genetic alterations of their own which confer resistance to FGFR inhibition. For instance, in the NCI-H1703 cell line, there are extremely high levels of PDGFB and PDGFC as well as copy-number amplification of their cognate receptor, PDGFRA (Supplementary Figure S4A). The PDGFR shares many downstream signaling molecules with the FGFR such as RAS, PI3K, ERK, and MYC [28,29]. A constitutively active PDGFR has commonly shared downstream molecules with the FGFR cascade, which ultimately induces resistance to FGFR inhibitors. In the case of the HCC15 cell line, it has high levels of EGF and mutated NRAS (Q61K) (Supplementary Figure S4B). This genomic condition can permanently activate downstream molecules, including RAF/MEK/ERK and PI3K/AKT, to strongly suppress P21 and activate MYC which ultimately promotes FGFR drug resistance. As a result, inhibition of FGFR did not affect proliferation in this network model. In NCI-H1781 (Supplementary Figure S4C) and NCI-H810 (Supplementary Figure S4D), high levels of EGF or PDGF ligands, respectively, for the corresponding cell lines regulate cellular responses to FGFR inhibitors. Taken together, we affirmed that resistant cell lines have specific driver genomic alterations from the RTKs or downstream molecules that are commonly shared with the FGFR signaling cascade and that can cause resistance to FGFR inhibitors. Therefore, we hypothesize that there could be an alternative regulatory mechanism other than the FGFR/ERK pathway that governs cellular phenotypes from the resistant lung cancer cell lines.

### 3.6. Differential Strategies and Mechanisms to Overcome FGFR Resistance

To validate our hypothesis and identify effective combinatorial targets to induce FGFR drug responses from the resistant cell lines, we calculated a synergy score between 51 possible pairs with FGFR inhibitor in our network model which is defined as follows [30]:

Synergy score = −[Drug response score of drug1 + drug2 − min(Drug response score of drug1, Drug response score of drug2)]

where Synergy score > 0 indicates synergy and Synergy score < 0 indicates antagonism.

As a result, none of the resistant cell lines, except NCI-H1703, showed synergistic drug response when GAB1 was co-inhibited with FGFR. This is mainly due to the positive feedback regulation between PI3K and GAB1 within the FGFR-AKT pathway that ultimately induces proliferation even with the blockade of the FGFR cascade. Thus, inhibition of GAB1 is necessary to lead the cells to become dependent on the FGFR-AKT pathway for FGFR drug sensitivity (Supplementary Figure S5).

**Figure 4:**
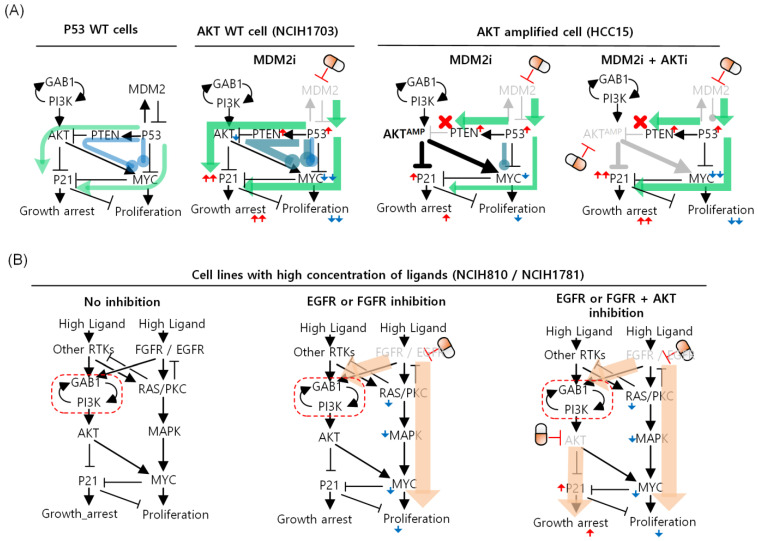
Identification of optimal targets specific to each resistant cell line. Identified targets and their specific mechanism in reducing proliferation and inducing growth arrest for drug sensitivity from each cell line-specific network model are shown. (**A**) MDM2 is shown as an optimal target to promote drug sensitivity in NCI-H1703 cell line. MDM2 and AKT are shown as optimal targets to promote drug sensitivity in HCC15 cell line. (**B**) EGFR and PDK1 are shown as optimal targets to promote drug sensitivity in NCI-H1781 cell line. FGFR and PDK1 are shown as optimal targets to promote drug sensitivity in NCI-H810 cell line.

To identify effective target(s) to reduce proliferation and induce growth arrest, we performed systematic single or double node perturbation analysis by using the selected 22 nodes with the available targeted drugs. In total, there were 253 perturbation conditions for each cell line-specific network model. Among the simulation results, we identified optimal target(s) with the highest value after the summation of highly ranked drug response and synergy score. For detailed information, please refer to Supplementary Table S1. In the case of HCC15, it has wildtype TP53 with gain-of-function mutation of RAS and mRNA overexpression of AKT meaning that both inhibition of MDM2 and AKT was necessary to reduce proliferation and induce growth arrest through P21 (Figure 4A). In contrast, NCI-H1703 has wildtype TP53 with no particular genomic alterations that would cause drug resistance. Thus, inhibition of MDM2 was sufficient to reduce proliferation and induce growth arrest. Moreover, the NCI-H1781 cell line has high levels of EGF ligand concentration and copy number amplification of EGFR and FGFR that means inhibition of both EGFR and AKT was necessary to block the signal transmission of both pathways. Similarly, the NCI-H810 cell line has high levels of EGF and PDGF ligand concentration and inhibition of both FGFR and AKT effectively induced cellular drug responses (Figure 4B). Our results showed that different cell types have their own complex regulatory mechanisms for regulating cellular phenotypes according to their genomic conditions.

## 4. Discussion

Conventional precision medicine in cancer has been focused on inhibiting genetically altered molecules within various signaling pathways as promising targets for anticancer drugs. However, the functional effects of these drugs have been reported to vary due to patient-specific genomic conditions which result in differential dynamics for each patient [31,32,33]. In this study, we developed a network model that can predict optimal targets for lung cancer by investigating cell type-specific genomic and transcriptomic alterations that can rewire network dynamics which subsequently determine their drug responses. We focused on FGFR inhibitors for lung cancer with a wide variety of interests in the field of anticancer drug discovery. First, we reconstructed a lung cancer Boolean network model by incorporating major signaling pathways associated with the FGFR signaling pathway to determine cellular phenotypes such as proliferation, growth arrest, and apoptosis. Furthermore, we generated seven cell line-specific Boolean models by applying their genomic and transcriptomic alterations from the CCLE cohort. By analyzing these cell line-specific lung cancer network models through computational simulation, we explicitly unraveled the mechanism of drug response of each model based on their genomic alterations.

Our study clearly highlights the influence of intercellular heterogeneity within signaling networks on predicting drug responses. In our simulation, the sensitive cell lines to FGFR inhibitors, including NCI-H1581, LK2 and NCI-H520, were highly dependent on the FGFR-ERK signaling due to their amplified expressions of FGFR and its cognate ligands. In contrast, inhibition of the PI3K/AKT signaling led the resistant cell lines to have partial responses to cell viability according to our simulation result (Supplementary Table S1). This is mainly due to the positive feedback regulation between PI3K and GAB1 within the FGFR-AKT pathway that ultimately activates the PI3K/AKT signaling even with the blockade of the FGFR cascade. Previous reports indicate that the PI3K signaling tends to be unresponsive to FGFR inhibition in most FGFR dependent-cancer cells, which is concordant with our simulation results [29,34]. Furthermore, our predicted results for FGFR-sensitive cell lines were in accordance with previous studies which are included in Supplementary Table S1.

Moreover, most FGFR inhibitor-resistant cell lines did not have any optimal targets inducing FGFR drug sensitivity, except for the NCI-H1703 cell line. For this cell line, due to PDGFR amplification, the activity of Grb2/Sos (GS) decreases slightly when only FGFR is inhibited. However, such inhibitory signaling cannot be further transmitted to their downstream molecules due to strong positive feedback between PI3K and GAB1. Thus, no phenotypic change for growth arrest of proliferation can be expected. Although the positive feedback between PI3K and GAB1 is turned off and partial inhibitory signal is transmitted to their downstream molecules through PI3K when GAB1 is inhibited, this inhibitory effect is very limited due to the amplified expression of EGFR and PDGFR. Thus, inhibitory effect can be transmitted to the downstream when both FGFR and GAB1 are inhibited in order to synergistically induce growth arrest and reduce proliferation in this cell line-specific model (Supplementary Figure S5).

To identify optimal targets that can induce therapeutic responses from FGFR-resistant cell lines, we further analyzed the network models of FGFR-resistant cell lines, including NCI-H1703, HCC15, and NCI-H1781. According to our simulation results, there are several alternative targets that can induce therapeutic responses in these cell lines. We have listed the targets in Supplementary Table S2. We identified that MDM2 and/or AKT inhibitors are alternative target(s) in NCI-H1703 or HCC15 cell lines, and AKT and EGFR inhibitors are alternative targets in the NCI-H1781 cell line, and these results were in accordance with previous studies (refer to Supplementary Table S2 for detailed references). Although there were alternative double targets in combination with higher drug response scores, there was no previous study to support our predicted results for these combinatory targets. Unfortunately, the scope of this study was limited to network reconstruction, target identification through computational analysis, and validation using previous studies. As a result, it is pertinent to further validate the predicted results by performing in vitro and in vivo experiments in our future studies. However, our results support the fact that the regulatory mechanisms for governing FGFR drug responses can vary between cell lines with different genomic backgrounds. Furthermore, we validated that the majority of our predicted targets were in accordance with previous studies and publicly available datasets. Therefore, it is necessary to systemically analyze the genomic profiles of patients to determine their specific targeted therapies according to the cellular dynamics that originate from their genomic environment.

Cancer is a heterogeneous disease with various genetic alterations that could be from different subgroups even within cancer patients from the same tissue. This can cause various limitations in precision medicine since cellular dynamics in each patient are different due to their distinct genomic alterations. Thus, it is imperative to study the effectiveness of each drug and its interactions with molecular components in a network model to predict drug responses and precisely identify optimal targets for each patient. Even with our effort to overcome these limitations, it is still hard to rationalize that our network can mimic the entire set of patients with diverse genomic backgrounds, including FGFR alterations, in the scope of this study. Therefore, it is essential to further extend our network model in order to represent the cellular dynamics of all lung cancer patients.

## 5. Conclusions

In summary, we reconstructed lung cancer specific network models that can mimic cellular responses to FGFR inhibitors using the corresponding experimental data from previous studies. Here, we showed that differential cellular responses to FGFR inhibitors can be determined by various genomic alterations in lung cancer cell lines. We also showed that there are differential mechanisms of FGFR drug responses from both sensitive and resistant cell lines. Moreover, we identified optimal targets for specific lung cancer cell lines that are resistant to FGFR inhibitors and elucidated the underlying mechanisms that induce therapeutic responses to alternative potential drug targets from each of the cell line-specific network models at a system level. It is noteworthy that, even though those cell lines have FGFR alterations, other drug candidates may induce better therapeutic responses depending on their genomic context. Therefore, our study demonstrates that the identification of drug targets for sensitivity using patient-specific network models could be a useful tool in precision medicine when used to study and analyze the dynamics of individualized interactions within the network model.

## Data Availability

Data is contained within the article or supplementary material.

## Supplementary Materials

The following supporting information can be downloaded at: https://www.mdpi.com/article/10.3390/biom12091197/s1, Figure S1: Creating functional genomic profile for reconstructing lung cancer network model to cell line-specific models; Figure S2: Drug prediction and genomic alterations from the selected lung cancer cell lines; Figure S3: Qualitative input–output relationships of the lung cancer network model; Figure S4: Responses to FGFR inhibitor from the resistant cell line-specific network models; Figure S5: Predicted drug synergy between FGFR inhibition and GAB1 inhibition in NCIH1703 cell line; Table S1: Agreement between in silico simulation results and experimental findings from published research about cellular responses to FGFR inhibition; Table S2: Top 5 single or double combinatorial targets for resistant cell lines.
