# Supplementary material for: Network Dynamics Caused by Genomic Alteration Determine the Therapeutic Response to FGFR Inhibitors for Lung Cancer
Source: Biomolecules. 2022 Aug 29;12(9):1197. doi: 10.3390/biom12091197 (PMC9496101; doi:10.3390/biom12091197)
Supplement: Supplementary file 1 [file biomolecules-12-01197-s001.zip › biomolecules-1833218-supplementary.pdf]

Title: Network dynamics caused by genomic alteration determine the therapeutic response to FGFR inhibitors for lung cancer

Jonghoon Lee, Sea Rom Choi, and Kwang-Hyun Cho*

Department of Bio and Brain Engineering, Korea Advanced Institute of Science and Technology (KAIST), Daejeon, Republic of Korea

*** Correspondence:**
Prof. Kwang-Hyun Cho. PhD.,
Department of Bio and Brain Engineering, KAIST,
291 Daehak-ro, Yuseong-gu, Daejeon 34141,
Republic of Korea.
Phone: +82-42-350-4325; Fax: +82-42-350-4310
E-mail: ckh@kaist.ac.kr

Supplementary Data

Supplementary figures

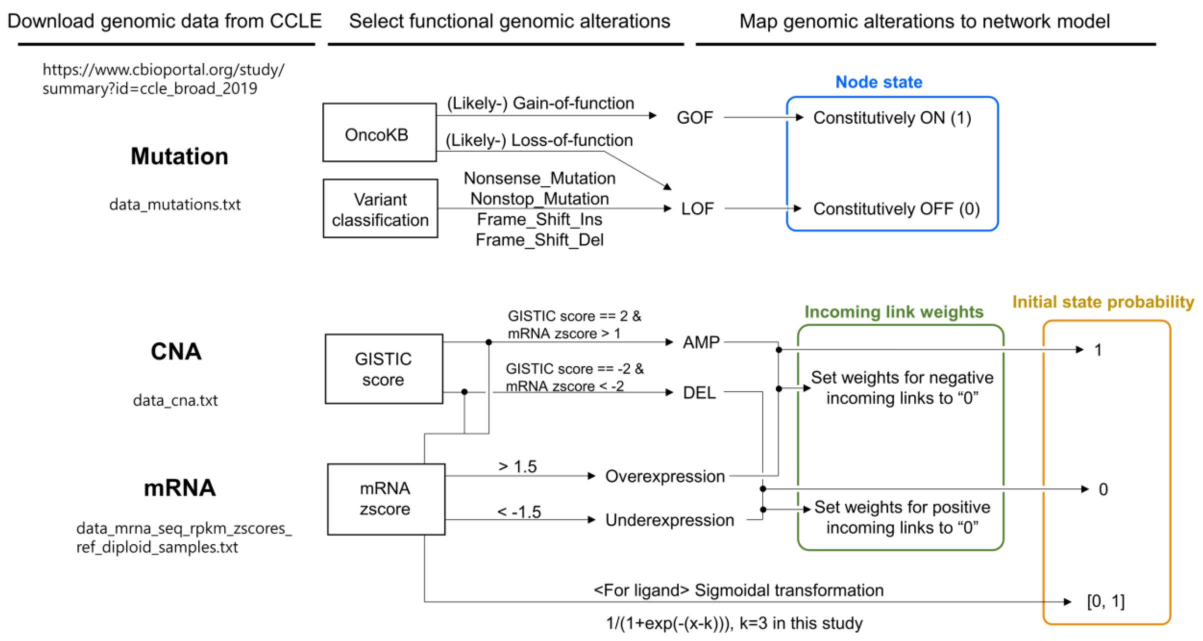

Figure S1. Creating functional genomic profile for reconstructing lung cancer network model to cell line-specific models.

To selectively choose the genes with functional genomic alteration, we analyzed the CCLE data of lung cancer cell lines. If a gene is included in our network model and has a somatic mutation with gain-of-function (GOF) from OncoKB databases, then we set the activity of this gene as ON (1). If a gene has a somatic mutation with loss-of-function (LOF), then we set the activity of this gene as OFF (0). In addition, variant_classification from maf file is annotated as 'Nonsense_Mutation', 'Nonstop_Mutation', 'Frame_Shift_Ins', or 'Frame_Shift_Del', we also set the activity of the gene as OFF. Z-score normalized mRNA expression data were downloaded from the CCLE cohort. For mRNA expression, we set a gene as overexpressed if its z-score is >-2 or underexpressed if its z-score is <-2. For CNA, we set a gene as amplified (AMP), if its GISTIC2 score is 2 and its mRNA expression is >1. Moreover, we set a gene as deletion (DEL), if its GISTIC2 score is -2.

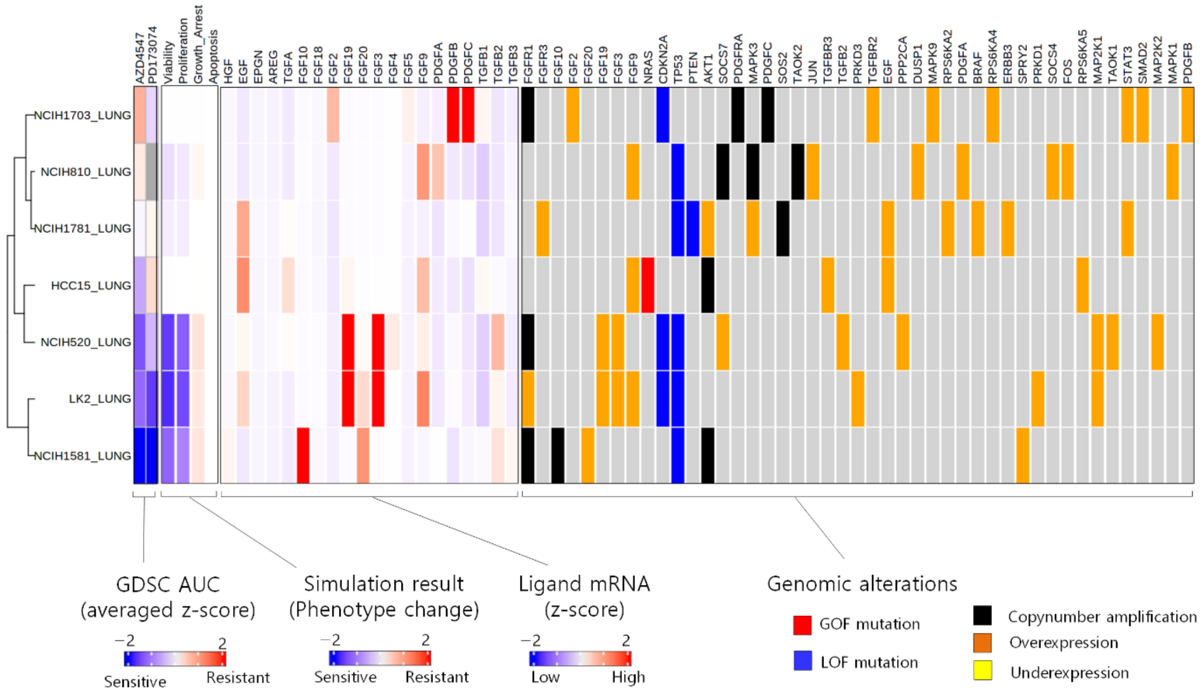

Figure S2. Drug prediction and genomic alterations from the selected lung cancer cell lines. Seven cell line-specific lung cancer network models with their responses to FGFR inhibitors are shown in the first column. Simulation results of predicted responses to FGFR inhibitors and genomic alteration information of each gene from the corresponding cell lines are shown in the next column.

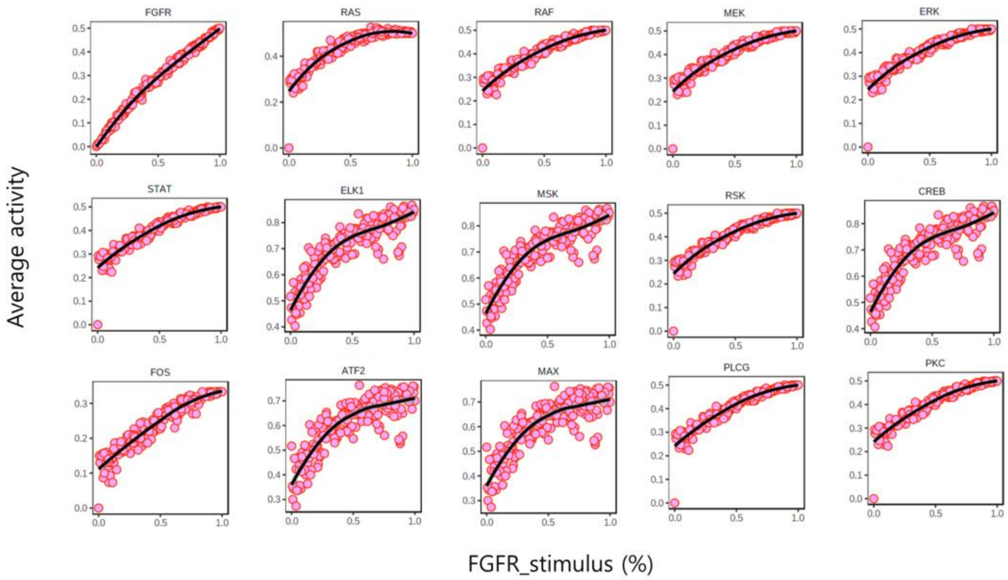

Figure S3. Qualitative input-output relationships of the lung cancer network model.

We validate our lung cancer network model by replicating physiological conditions of lung cancer cells through qualitative computational simulation. The simulation results after inducing EGF exhibited a positive relationship with its downstream nodes, including ERK, AKT, S6K, and MYC. The results indicate that our network model can reflect the biological properties of lung cancer cells.

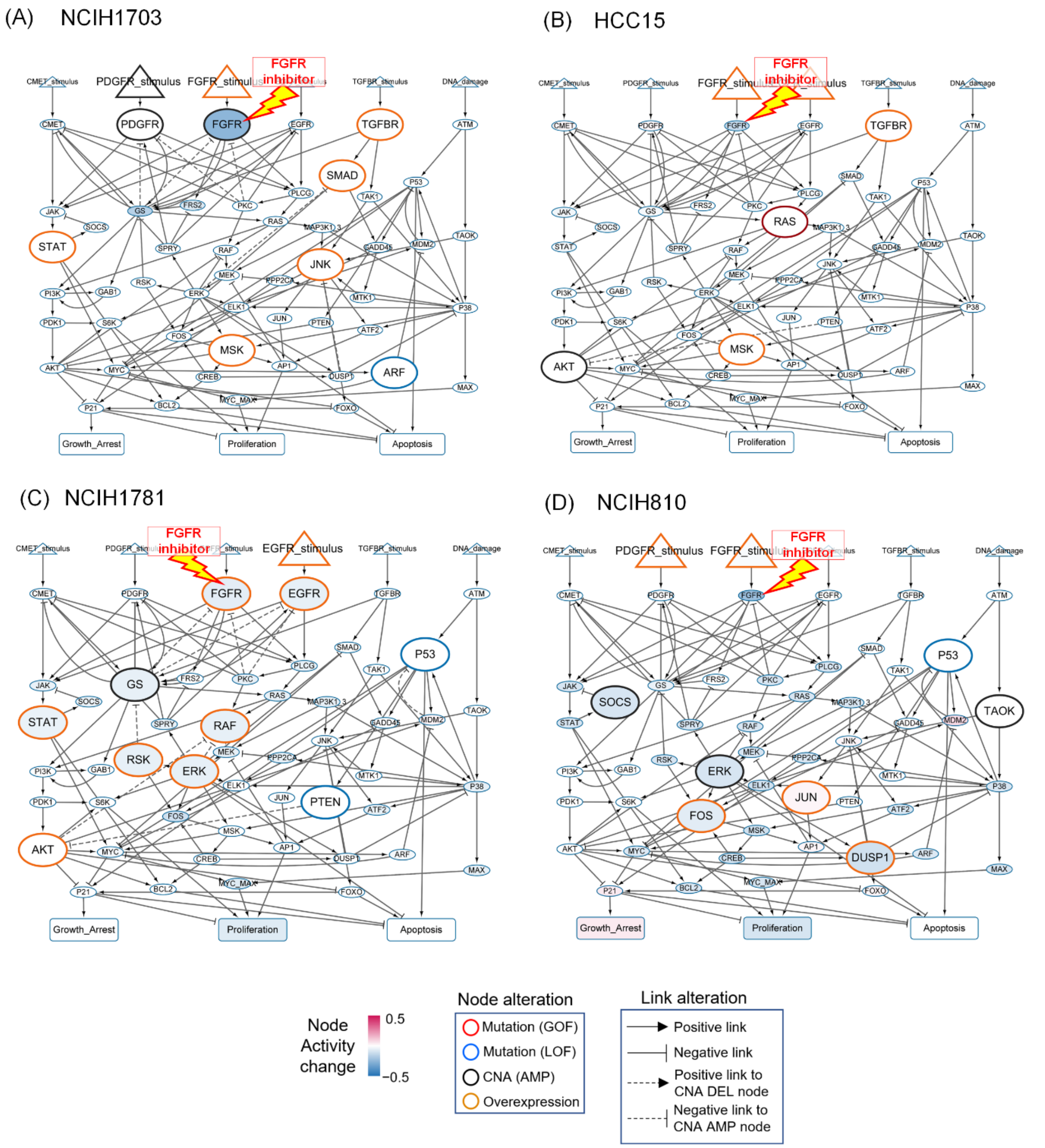

Figure S4. Responses to FGFR inhibitor from the resistant cell line-specific network models. The average activity changes of FGFR, its downstream molecules, and changes in phenotype nodes before and after the drug treatment are shown for (A) NCI-H1703, (B) HCC15, (C) NCI-H1781, and (D) NCI-H810. Red open circle represents gain-of-function mutation, blue open circle represents loss-of-function mutation, black open circle represents copy number amplification, and yellow open circle represent overexpression. Solid arrow indicates positive link whereas blunted-end head arrow indicates negative link. Dotted arrow indicates positive link to node with CNA deletion whereas dotted arrow with blunted-end indicates negative link to node with CNA amplification.

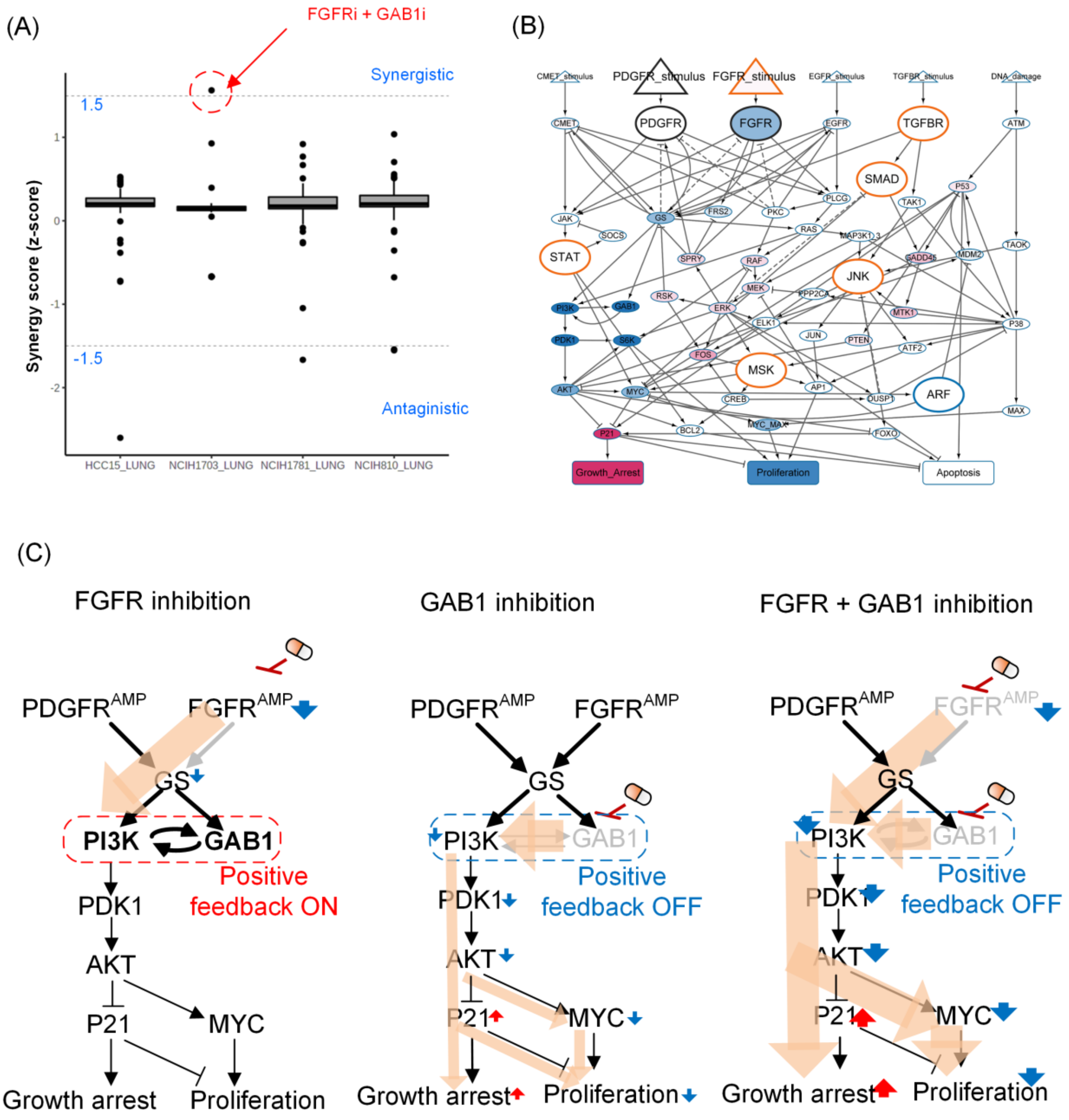

Figure S5. Predicted drug synergy between FGFR inhibition and GAB1 inhibition in NCIH1703 cell line.

(A) Identification of optimal drug targets from the FGFR inhibitor-resistant cell lines, HCC15, NCIH1703, NCIH1781, and NCIH810, by using their synergy scores. We define synergy z-score higher than 1.5 is considered synergistic and lower than -1.5 is considered antagonistic. Only NCIH1703 cell line has a synergistic combinatory target, FGFR and GAB1, to induce FGFR drug sensitivity. (B) Changes in node activities from the NCI-1703 specific network model are shown after both FGFR and GAB1 are inhibited. Node color with red indicates increasing node activity whereas blue indicates decreasing node activity. Higher the intensity show, greater the activity change there is. (C) Due to PDGFR amplification, the activity of Grb2/Sos (GS) decreases slightly when only FGFR is inhibited. However, such inhibitory

signaling cannot be further transmitted to their downstream molecules due to strong positive feedback between PI3K and GAB1. Thus, no phenotypic change for growth arrest of proliferation can be expected. Although the positive feedback between PI3K and GAB1 is turned off and partial inhibitory signal is transmitted to their downstream molecules through PI3K when GAB1 is inhibited, this inhibitory effect is very limited due to the amplified expression of EGFR and PDGFR. Therefore, inhibitory effect can be transmitted to the downstream when both FGFR and GAB1 are inhibited in order to synergistically induce growth arrest and reduce proliferation in this cell line-specific model.

Table S1. Agreement between in silico simulation results and experimental findings from published research about cellular responses to FGFR inhibition.

Cell line	Response to FGFRi	Readout (Experiment)	Node (*in silico*)	Explained by the model	Reference (PMID)
NCIH1581	decreased	p-FGFR1	FGFR	yes	27401245, 26549034
		p-FRS2a196/439	FRS	yes	27401245, 28968756
		p-Erk	ERK	yes	27401245, 26549034, 28968756
		p-PLCgamma	PLCG	yes	27401245
		c-Myc	MYC	yes	27401245
		p-Stat3	STAT	no	27401245
		Cell viability	Viability score	yes	27401245
	increased	p21	P21	yes	27401245
	constant	p-AKT	AKT	yes	27401245, 26549034, 28968756
		p-S6	S6K	yes	26549034
NCIH520	decreased	p-FGFR1	FGFR	yes	27401245, 26549034
		p-FRS2a196/439	FRS	yes	27401245, 28968756
		p-Erk	ERK	yes	27401245, 26549034, 28968756
		p-PLCgamma	PLCG	yes	27401245
		c-Myc	MYC	yes	27401245
		p-Stat3	STAT	no	27401245
		Cell viability	Viability score	yes	27401245
	increased	p21	P21	yes	27401245
	constant	p-AKT	AKT	yes	27401245, 26549034, 28968756
		p-S6	S6K	yes	26549034
NCIH1703	constant	p-PDGFR	PDGFR	yes	26549034
		p-FRS2	FRS2	yes	26549034, 28968756
		p-Erk	ERK	yes	26549034, 28968756
		p-Akt	AKT	yes	26549034, 28968756
		Cell viability	Viability score	yes	26549034

Table S2. Top 5 single or double combinatorial targets for resistant cell lines. We performed single or double node perturbation analysis and selected top 5 simulation results for each cell line-specific network model. For double node perturbation, we rank the simulation results of drug response score and synergy score, and add them up according to each perturbation. The row shaded in yellow is selected optimal targets for the corresponding cell line. The rows shaded in green are single target that has partial responses in promoting drug sensitivity that were commonly found from the entire resistant cells.

Cell	Inhibition	Drug_Response_Score	Synergy_Score	PMID
HCC15_LUNG	MDM2i & JAKi	-1.458325053	0.823200977	NA
	AKTi & MDM2i	-1.458325053	0.441328268	NA
	MDM2i & PDK1i	-1.458325053	0.435894683	NA
	MDM2i & PI3Ki	-1.458325053	0.430767615	NA
	MDM2i & GSi	-1.208094639	0.561476163	NA
	PI3Ki	-1.027557439	NA	26119936 (PI3Ki)
	PDK1i	-1.02243037	NA	26119936 (PI3Ki)
	AKTi	-1.016996785	NA	26119936 (PI3Ki)
	GSi	-0.646618476	NA	NA
	MDM2i	-0.635124076	NA	NA
NCIH1703_LUNG	MDM2i	-1.867625899	NA	33710818 (MDM2i)
	AKTi & JAKi	-1.867625899	0.224292432	NA
	JAKi & PI3Ki	-1.867625899	0.224292432	NA
	PDK1i & JAKi	-1.867625899	0.224292432	NA
	PDK1i & PDGFRi	-1.64752947	0.24196023	NA
	AKTi & PDGFRi	-1.646193751	0.240393108	NA
	JAKi	-1.643333467	NA	22319590 (JAK2i) 31595832 (STAT3i)
	AKTi	-1.405800642	NA	11358816 (PI3Ki)
	PI3Ki	-1.405634793	NA	11358816 (PI3Ki)
	PDK1i	-1.40556924	NA	11358816 (PI3Ki)
NCIH1781_LUNG	PDK1i & EGFRi	-0.831649538	0.199804679	NA
	EGFRi & PI3Ki	-0.828710032	0.200600209	NA
	AKTi & EGFRi	-0.812124674	0.252109642	NA
	AKTi & RAFi	-0.653757965	0.239795868	NA
	AKTi & MEKi	-0.644913712	0.230951615	NA
	PDK1i	-0.631844858	NA	NA
	PI3Ki	-0.628109824	NA	NA

	S6Ki	-0.593854919	NA	NA
	EGFRi	-0.560015032	NA	NA
	AKTi	-0.413962097	NA	NA
NCIH810_LUNG	AKTi & FGFRi	-0.378805483	0.03969501	NA
	PDK1i & FGFRi	-0.378081478	0.024649247	NA
	FGFRi & PI3Ki	-0.376098703	0.023364029	NA
	AKTi & JAKi	-0.361491431	0.022380958	
	AKTi & S6Ki	-0.357226947	0.018116474	
	PDK1i	-0.353432231	NA	
	PI3Ki	-0.352734675	NA	
	AKTi	-0.339110472	NA	
	FGFRi	-0.13717549	NA	
	GSi	-0.127930193	NA	
